# Supplementary material for: More efficient and inclusive time-to-event trials with covariate adjustment: a simulation study
Source: Trials. 2023 Jun 6;24:380. doi: 10.1186/s13063-023-07375-0 (PMC10245605; doi:10.1186/s13063-023-07375-0)
Supplement: Supplementary file 1 — Additional file 1: Table S1. Description of simulation parameters used for parametric simulations of the time-to-event model. Figure S1. Evolution of $R_{obs}^2$Robs2 as a function of C-index, cumulative incidence, treatment effect, Weibull shape w and drop-out rate d. A: θ = 0.7, B: θ = 0.4. Figure S2. Power curves resulting from adjustment with clinical variables only (tumor staging and ECOG score) or with the additional deep learning HCCnet covariate. Covariates are sampled from the HCC patients of the TCGA dataset. Figure S3. Relationships between proposed $R^2$R2 measures and the reduction of sample size provided by covariate adjustment $R_{obs}^2$Robs2 over the grid of parameters described in Table S1. Each point on each panel corresponds to a unique combination of values for the five parameters in Table S1. Each unique combination of parameters has therefore eight corresponding $R^2$R2 measures. The notations for different measures of [file 13063_2023_7375_MOESM1_ESM.docx]

# Supplementary Materials for: More efficient and inclusive time-to-event trials with covariate adjustment: a simulation study

Raphaëlle Momal^1^*, Honghao Li^1^*, Paul Trichelair^1^, Michael G.B. Blum^1^, Félix Balazard^1$^

1. Owkin Inc., New York, USA

*: contributed equally

$: corresponding author felix.balazard@owkin.com

#### Supplementary Tables

| \| Quantity \| Notation \| Values \| \| --- \| --- \| --- \| \| Cumulative incidence \| $\Lambda$ \| {0.1, 0.2, 0.3, 0.4, 0.5, 0.6, 0.7, 0.8, 0.9} \| \| C-index \| $C$ \| {0.55, 0.65, 0.75, 0.85} \| \| Weibull shape \| $w$ \| {0.5, 1, 1.5} \| \| Drop-out rate \| $d$ \| {0.01, 0.1} \| \| Treatment hazard ratio \| $hr$ \| {0.4, 0.7} \| |
| --- | --- | --- | --- | --- | --- | --- | --- | --- | --- | --- | --- | --- | --- | --- | --- | --- | --- | --- |
| Table S1: Description of simulation parameters used for parametric simulations of the time-to-event model. |

#### Supplementary Figures

| 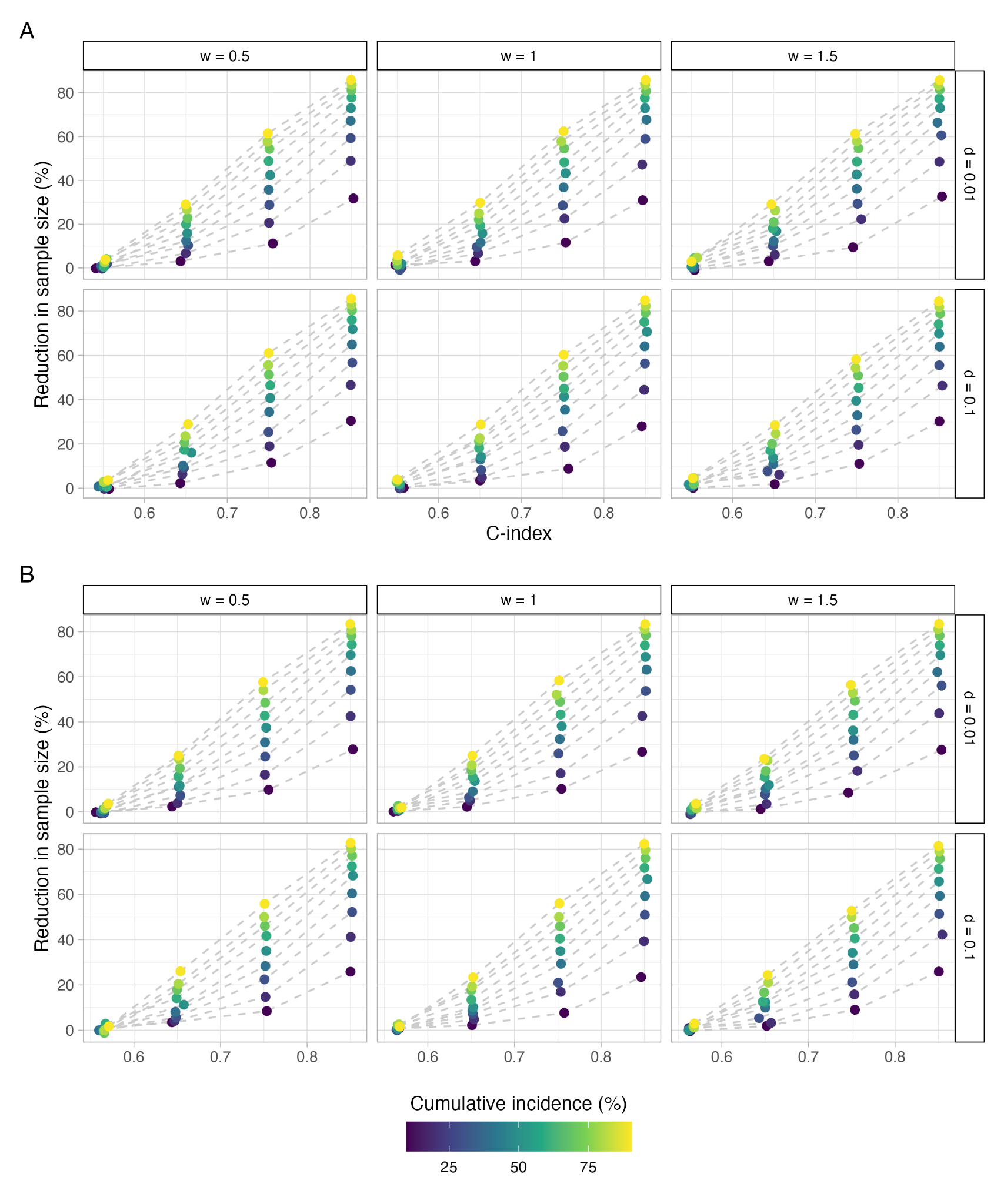 |
| --- |
| Figure S1: Evolution of $R_{\mathsf{obs}}^{2}$ as a function of C-index, cumulative incidence, treatment effect, Weibull shape *w* and drop-out rate *d*. A: $\theta=0.7$, B: $\theta=0.4$. |


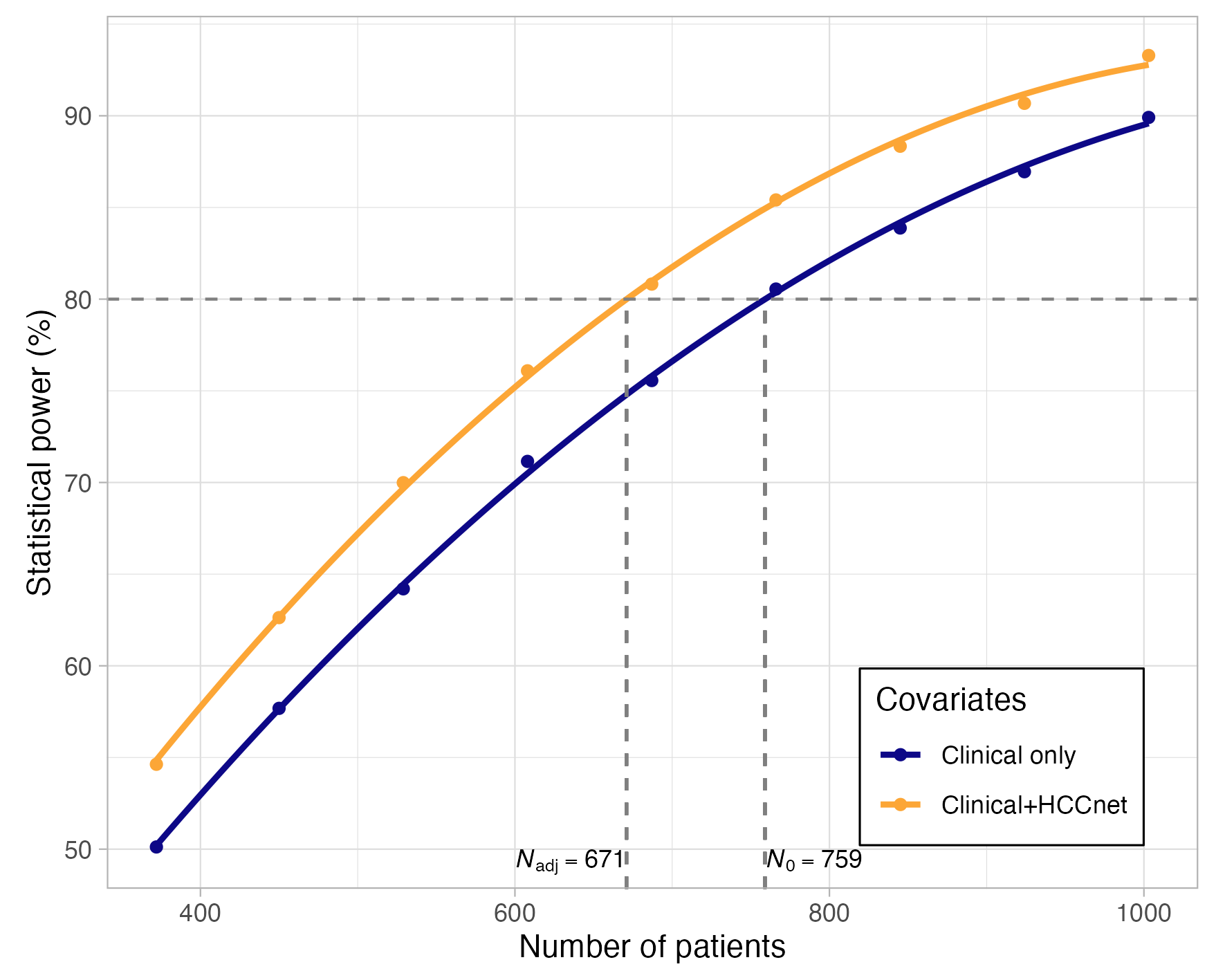


Figure S2: Power curves resulting from adjustment with clinical variables only (tumor staging and ECOG score) or with the additional deep learning HCCnet covariate. Covariates are sampled from the HCC patients of the TCGA dataset.


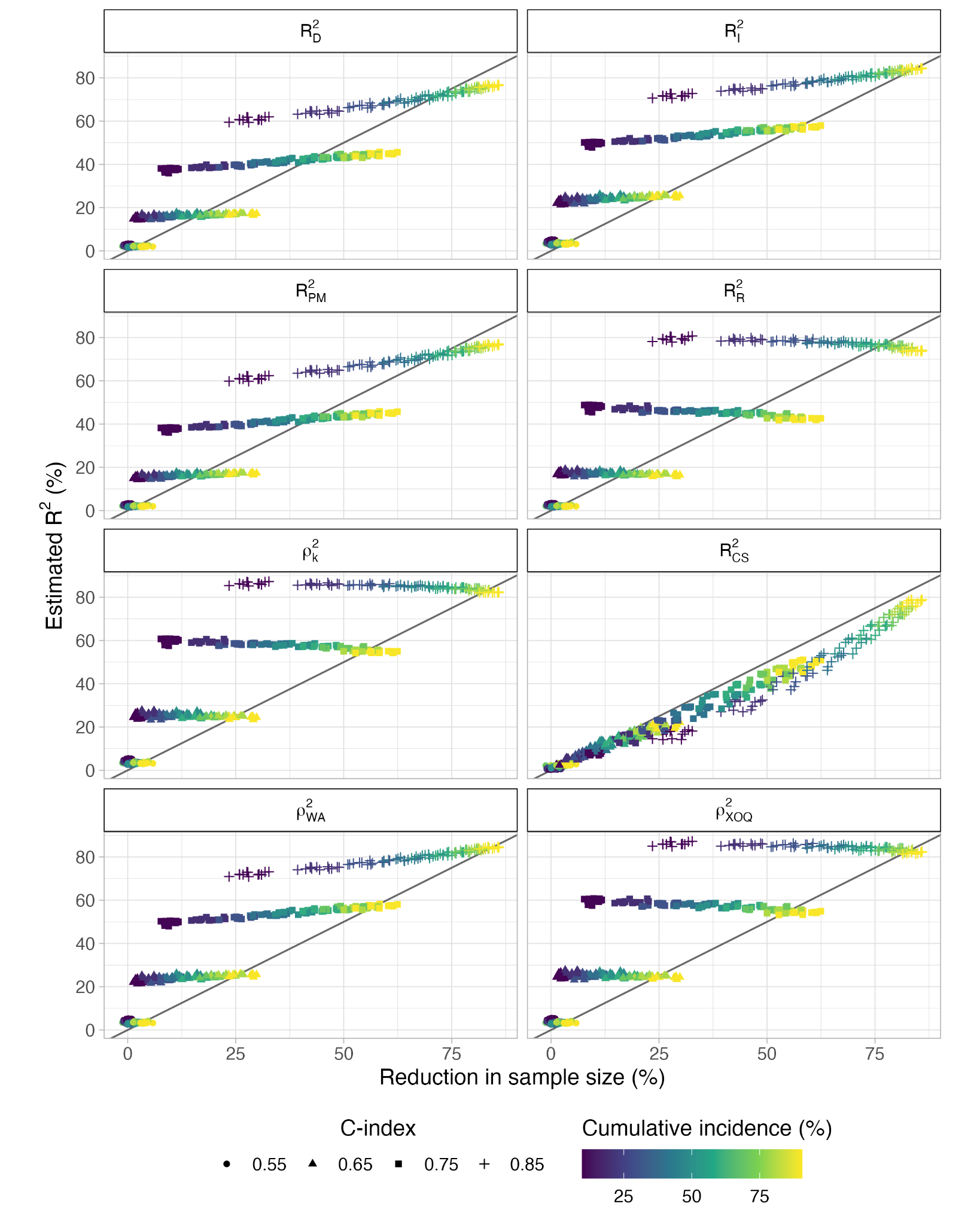


Figure S3: Relationships between proposed $R^{2}$ measures and the reduction of sample size provided by covariate adjustment $R_{\mathsf{obs}}^{2}$ over the grid of parameters described in table S1. Each point on each panel corresponds to a unique combination of values for the five parameters in Table S1. Each unique combination of parameters has therefore eight corresponding $R^{2}$ measures. The notations for different measures of $R^{2}$ follow mainly [28]. $R_{CS}^{2}$ is the Cox-Snell $R_{CS}^{2}$ which is related to the likelihood ratio between the model of interest and a null model. $\rho_{k}^{2}$ is a variation of $R_{CS}^{2}$. $R_{D}^{2}$ is a transformation of the $D$ measure. $R_{I}^{2}$ is a variation of $R_{D}^{2}$. $R_{PM}^{2}$ is a measure related to the squared Pearson correlation between the logarithm of transformed survival time and the term $\beta X$. $R_{R}^{2}$ is the measure proposed by Royston in the same paper of reference [28]. $\rho_{WA}^{2}$ is an approximated version of a more complex measure related to the Weibull model. $\rho_{XOQ}^{2}$ is a measure named after the authors of the paper: Xu, O’Quigley [30].
